# *Cissus verticillata* Extract Decreases Neuronal Damage Induced by Oxidative Stress in HT22 Cells and Ischemia in Gerbils by Reducing the Inflammation and Phosphorylation of MAPKs

**DOI:** 10.3390/plants10061217

**Published:** 2021-06-15

**Authors:** Woosuk Kim, Hyun Jung Kwon, Hyo Young Jung, Soon-Sung Lim, Beom-Goo Kang, Yong-Bok Jo, Dong-Sool Yu, Soo Young Choi, In Koo Hwang, Dae Won Kim

**Affiliations:** 1 Department of Biomedical Sciences, and Research Institute for Bioscience and Biotechnology, Hallym University, Chuncheon 24252, Korea; wskim0503@konkuk.ac.kr (W.K.); donuts25@hallym.ac.kr (H.J.K.); sychoi@hallym.ac.kr (S.Y.C.); 2 Department of Anatomy, College of Veterinary Medicine, Veterinary Science Research Institute, Konkuk University, Seoul 05030, Korea; 3 Department of Biochemistry and Molecular Biology, Research Institute of Oral Sciences, College of Dentistry, Gangneung-Wonju National University, Gangneung 25457, Korea; 4 Department of Veterinary Medicine, Institute of Veterinary Science, Chungnam National University, Daejeon 34134, Korea; hyjung@cnu.ac.kr; 5 Department of Food Science and Nutrition, Institute of Korean Nutrition, Hallym University, Chuncheon 24252, Korea; limss@hallym.ac.kr; 6 Department of Biochemistry, College of Medicine, Hallym University, Chuncheon 24252, Korea; kbgda87@naver.com; 7 Department of Convergence Technology, Graduate School of Venture, Hoseo University, Seoul 06724, Korea; korjyb59@korea.ac.kr; 8 Department of Venture Management Graduate School of Venture, Hoseo University, Seoul 06724, Korea; sdt501@korea.ac.kr; 9 Department of Anatomy and Cell Biology, College of Veterinary Medicine and Research Institute for Veterinary Science, Seoul National University, Seoul 08826, Korea

**Keywords:** *Cissus verticillata* extract, oxidative stress, ischemia, inflammation, MAPK

## Abstract

In the present study, we examined the effects of *Cissus verticillata* leaf extracts (CVE) against hydrogen peroxide (H_2_O_2_)- and ischemia-induced neuronal damage in HT22 cells and gerbil hippocampus. Incubation with CVE produced concentration-dependent toxicity in HT22 cells. Significant cellular toxicity was observed with >75 μg/mL CVE. CVE treatment at 50 μg/mL ameliorated H_2_O_2_-induced reactive oxygen species formation, DNA fragmentation, and cell death in HT22 cells. In addition, incubation with CVE significantly mitigated the increase in Bax and decrease in Bcl-2 induced by H_2_O_2_ treatment in HT22 cells. In an in vivo study, the administration of CVE to gerbils significantly decreased ischemia-induced motor activity 1 d after ischemia, as well as neuronal death and microglial activation 4 d after ischemia, respectively. CVE treatment reduced the release of interleukin-1β, interleukin-6, and tumor necrosis factor-α 6 h after ischemia. Furthermore, CVE treatment significantly ameliorated ischemia-induced phosphorylation of c-Jun N-terminal kinase, extracellular signal-regulated kinase 1/2, and p38. These results suggest that CVE has the potential to reduce the neuronal damage induced by oxidative and ischemic stress by reducing the inflammatory responses and phosphorylation of MAPKs, suggesting that CVE could be a functional food to prevent neuronal damage induced by ischemia.

## 1. Introduction

Hydrogen peroxide (H_2_O_2_) is a well-known reactive oxygen species (ROS) and causes oxidative stress in various cell lines, including HT22 hippocampal cells [1,2,3]. Mongolian gerbils (*Meriones unguiculatus*) are widely used as a transient ischemic model due to the absence of a posterior communicating artery and as they require less equipment for operating compared to the focal ischemic model [4]. Among brain regions, the hippocampus, thalamus, and neocortex are highly susceptible to transient forebrain ischemia in gerbils [5,6]. Damage to the hippocampus causes hyperactivity and memory impairment due to hippocampal neuronal damage [7,8]. Ischemia is caused by an insufficient blood supply which does not meet the metabolic requirements in the brain, leading to brain tissue damage [9]. In particular, interruption of blood vessels and reperfusion enormously increases the formation of ROS because of the high content of unsaturated fatty acids in neurons [10]. Increased ROS react with unsaturated fatty acids in membranes and DNA in the nucleus and causes the neuronal damage. Damaged neurons trigger the activation of microglia and subsequent generation of pro-inflammatory cytokines, such as interleukin (IL)-1β, IL-6, and tumor necrosis factor-alpha (TNF-α) [11,12,13]. The released cytokines recruit immune cells in the affected area and finally release cytotoxins, including metalloproteinases [14,15]. Cell death mechanisms have been widely elucidated [16,17], but few therapeutics have been approved for the treatment of ischemia.

The *Vitaceae* family has been widely used in oriental medicine because of its anti-inflammatory and antioxidant effects [18,19]. The *Vitaceae* family consists of four genera. The genus *Vitis* is the best-known plant in this family. Previous studies have demonstrated the neuroprotective effects of *Vitis* species against ischemic damage [20,21,22,23]. In a previous study, we, together with our colleagues, demonstrated that grape seed extract reduced the DNA damage induced by ischemia in the gerbil hippocampus [20]. In contrast, the genus *Cissus* consists of approximately 350 species in tropical and subtropical areas. *C. quadrangularis* activity in reducing obesity and osteoarthritis has been described [24,25]. *C. verticillata* (also known as *C. sicyoides* or possum grape vine) is native to Central America. It is cultivated as a medicinal plant because of its anti-diabetic [26,27], anti-inflammatory [28], and anxiolytic [29] effects. However, few studies have investigated the effects of *C. verticillata* extract (CVE) on ischemic damage in the brain.

In the present study, we screened the effects of the CVE on cell damage in the HT22 hippocampal cells lines subjected to H_2_O_2_ and gerbil model of cerebral ischemia. In addition, we elucidated the probable mechanisms of CVE against oxidative and ischemic damage based on oxidative stress and inflammatory responses.

## 2. Results

### 2.1. Determination of Optimal Concentration

**Figure 1:**
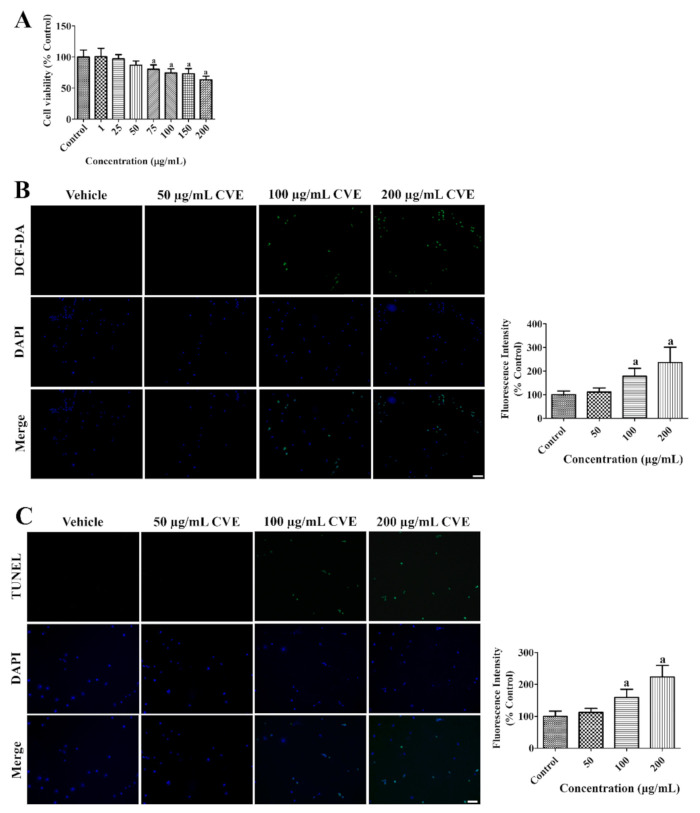
*Cissus verticillata* extract (CVE) has toxic effects in high concentration in HT22 cells. (**A**) Cellular toxicity of CVE was confirmed by WST-1 assay to determine the optimal concentration with minimal toxicity. (**B**) ROS formation and (**C**) DNA fragmentation was confirmed by DCF fluorescence and TUNEL staining, respectively, in the control, 50, 100, and 200 μg/mL CVE-treated group for 60 min. Scale bar = 50 μm. Fluorescent intensities are measured using an enzyme-linked immunosorbent assay (ELISA) reader. Data are presented as mean value ± standard deviation and were analyzed using one-way analysis of variance (ANOVA) followed by Bonferroni’s post hoc test (^a^ *p* < 0.05, significantly different from the control group).

The toxicity of CVE was assessed to determine the optimal concentration to prevent death in HT22 cells by 2′,7′-dichlorofluorescein (DCF) fluorescence, terminal deoxynucleotidyl transferase dUTP nick end labeling (TUNEL) staining, and WST-1 assays. Incubation with CVE for 60 min showed no or less DCF and TUNEL fluorescence as well as cell death in HT22 cells at a concentration of 50 μg/mL. However, cell viability was significantly decreased concentration-dependently and about 63.3% of cells were found after incubation with 200 μg/mL CVE (Figure 1A). Treatment with 100–200 μg/mL CVE significantly increased DCF and TUNEL fluorescence concentration-dependently in HT22 cells by 236.0% and 223.3% of control group, respectively (Figure 1B,C).

### 2.2. Effects of CVE on H_2_O_2_-Induced Oxidative Stress in HT22 Cells

**Figure 2:**
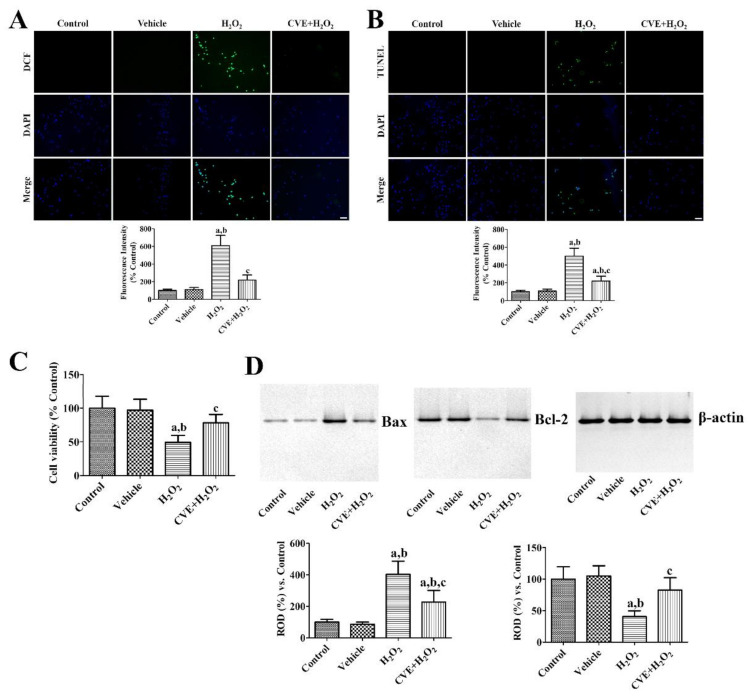
*Cissus verticillata* extract (CVE) reduces H_2_O_2_-induced oxidative damage in HT22 cells. (**A**) ROS formation and (**B**) DNA fragmentation was visualized by DCF and TUNEL staining, respectively, in the control, vehicle (PBS)-treated group, H_2_O_2_-treated (H_2_O_2_) group, and H_2_O_2_-treated group with 50 μg/mL CVE (CVE + H_2_O_2_). Scale bar = 50 μm. Fluorescent intensities are measured using an enzyme-linked immunosorbent assay (ELISA) reader. (**C**) Cellular damage and (**D**) apoptotic and anti-apoptotic markers are assessed by WST-1 assay and western blot for Bax and Bcl-2, respectively. Western blot data are quantified and normalized to β-actin levels. Data are presented as mean value ± standard deviation and were analyzed using one-way analysis of variance (ANOVA) followed by Bonferroni’s post hoc test (^a^ *p* < 0.05, significantly different from the control group; ^b^ *p* < 0.05, significantly different from the vehicle group; ^c^ *p* < 0.05, significantly different from the H_2_O_2_ group).

The effects of CVE against H_2_O_2_-induced oxidative stress were validated by DCF fluorescence, TUNEL staining, and WST-1 assays. In the control and vehicle-treated groups, DCF and TUNEL fluorescence were very weak in HT22 cells, whereas in the H_2_O_2_-treated group strong DCF and TUNEL fluorescence were observed in HT22 cells. In addition, the DCF and TUNEL fluorescent intensities were significantly increased to 608.1% and 499.9%, respectively, compared to the respective control group. In the 50 μg/mL CVE and H_2_O_2_-treated (CVE + H_2_O_2_) group, a few cells showed DCF and TUNEL fluorescence in HT22 cells. Their fluorescence intensity was significantly lower than that of the respective H_2_O_2_-treated group (216.8% and 220.8% of the control group, respectively; Figure 2A,B).

Cell viability was significantly decreased in the H_2_O_2_-treated group to 49.1% of control group. In the CVE + H_2_O_2_ group, cell viability was significantly greater (78.3% of the control group) than in the H_2_O_2_-treated group (Figure 2C).

Bax and Bcl-2 protein levels were similar between the control and vehicle-treated groups. However, in the H_2_O_2_-treated group, Bax protein levels were dramatically increased to 403.4% of the control group 6 h after H_2_O_2_ treatment, while Bcl-2 levels were significantly lower (40.6%) than the control group. In the CVE + H_2_O_2_ group, the changes in Bax and Bcl-2 levels were significantly reduced to 226.7% and 82.7% of the control group, respectively, compared to the H_2_O_2_-treated group (Figure 2D).

### 2.3. Effects of CVE on Motor Activity and Cell Death in the Hippocampus after Ischemia

**Figure 3:**
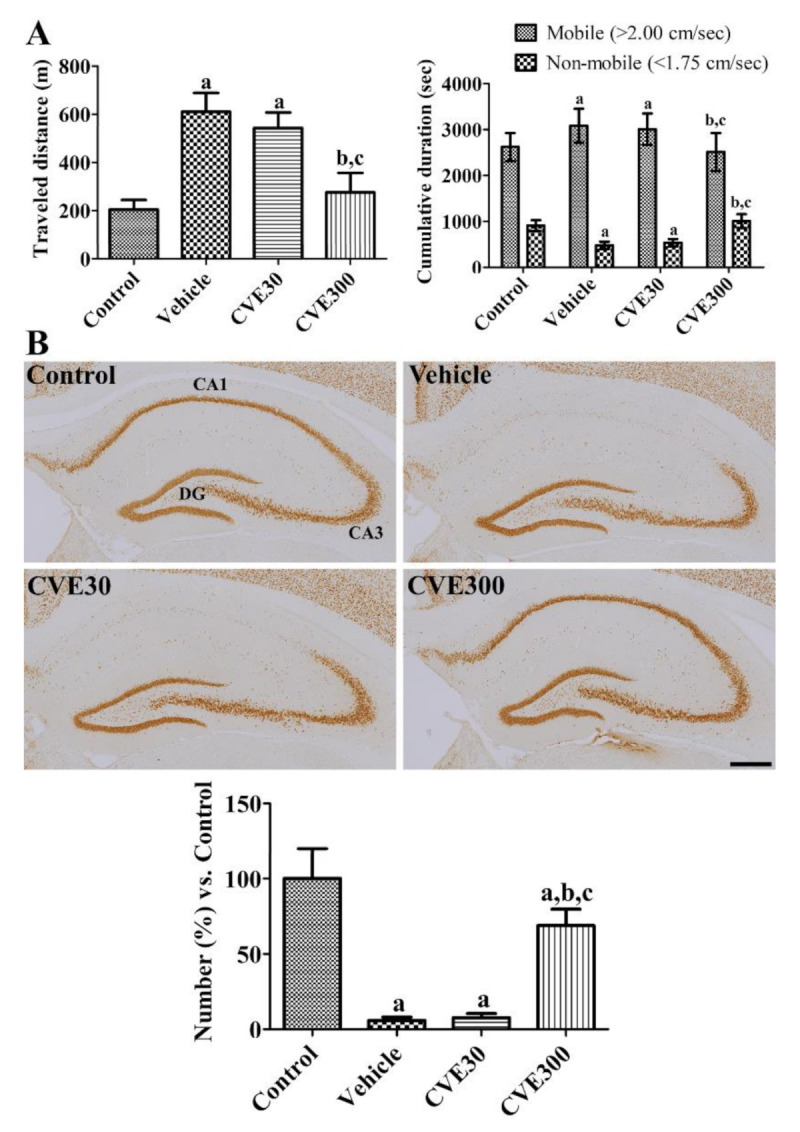
*Cissus verticillata* extract (CVE) ameliorates ischemia-induced hyperlocomotor activity and neuronal death in gerbils. (**A**) One day after ischemia, locomotor activity was analyzed based on travel distance and cumulative duration in sham-operated (control), ischemia-induced vehicle-treated (vehicle), ischemia-induced 30 mg/kg CVE-treated (CVE30), and ischemia-induced 300 mg/kg CVE-treated (CVE300) groups (*n* = 10 per group). (**B**) Four days after ischemia, the hippocampi were immunostained with NeuN antibody to detect survived neurons. Scale bar = 400 μm. The number of NeuN-immunoreactive neurons in the hippocampal CA1 region is presented as a percentile value compared to control group (*n* = 5 per group). (**A**,**B**) Data are presented as mean value ± standard deviation and were analyzed using one-way ANOVA followed by Bonferroni’s post hoc test (^a^ *p* < 0.05, significantly different from the control group; ^b^ *p* < 0.05, significantly different from the vehicle group; ^c^ *p* < 0.05, significantly different from the CVE30 group).

In the vehicle- and CVE30-treated ischemic groups, the travel distance was significantly longer 1 d after ischemia compared to that in the control group (298.1% and 265.4% that of the control group, respectively). Time duration in mobile phases was significantly increased in the vehicle- and CVE30-treated ischemic groups, while it was significantly decreased in non-mobile phases. In the CVE300-treated ischemic group, the travel distance was significantly decreased to 134.8% of the control group compared to the vehicle- and CVE30-treated ischemic groups. In addition, the time spent in the mobile and non-mobile phases was shorter and longer, respectively, in the CVE300-treated ischemic group than in the vehicle- and CVE30-treated ischemic groups (Figure 3A).

In the control group, hematoxylin and eosin stained cells were morphologically intact in the hippocampal CA1 and CA2 region. In the vehicle- and CVE30-treated ischemic groups, there were aggregations of hematoxylin stained nuclei in the stratum pyramidale of CA1 region, but not in the CA2 region. In the CVE300-treated ischemic group, some hematoxylin stained nuclei were found in the stratum pyramidale (Supplementary Figure S1). 

The mature nuclei were confirmed by immunohistochemical staining for NeuN in the hippocampus. In the control group, neuronal nuclei (NeuN)-immunoreactive neurons were detected in the hippocampus. In the vehicle- and CVE30-treated ischemic groups, NeuN-immunoreactive neurons were abundant in the hippocampal CA3 region and dentate gyrus, while a few neurons were found in the hippocampal CA1 region. The number of NeuN-immunoreactive neurons in these groups was 5.8% and 7.7% of that in the control group, respectively. In the CVE300-treated ischemic group, many NeuN-immunoreactive neurons were observed in the hippocampus, including the CA1 region. In this group, the number of NeuN-immunoreactive neurons in the CA1 region was significantly increased to 68.8% in the control group compared to the vehicle- and CVE30-treated ischemic groups (Figure 3B).

### 2.4. Mechanisms of CVE against Ischemic Damage in Gerbils

**Figure 4:**
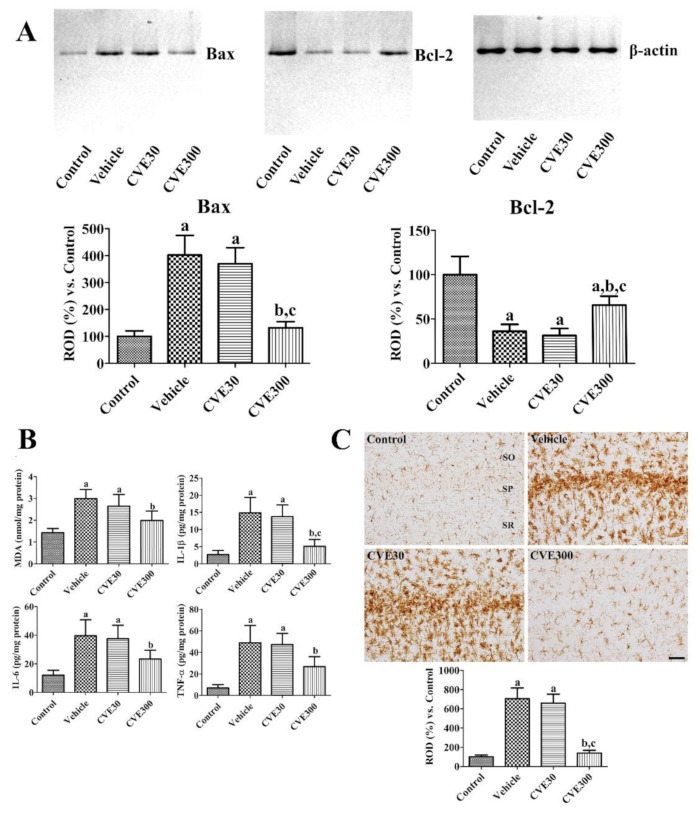
*Cissus verticillata* extract (CVE) ameliorates ischemia-induced apoptosis/anti-apoptosis, lipid peroxidation, pro-inflammatory cytokine releases, and microglial activation. (**A**) Three days after ischemia, apoptosis and anti-apoptosis were analyzed in the hippocampus using western blot for Bax and Bcl-2. (**B**) Six hours after ischemia, lipid peroxidation and pro-inflammatory cytokines were analyzed based on ELISA assay for MDA, IL-1β, IL-6, and TNF-α in the gerbil hippocampus of control, vehicle, CVE30, and CVE300 groups (*n* = 5 per group). (**C**) Four days after ischemia, the hippocampi were immunostained with Iba-1 antibody to detect microglia in the hippocampal CA1 region. SO, stratum oriens; SP, stratum pyramidale; SR, stratum radiatum. Scale bar = 50 μm. Optical density was analyzed and relative optical density (ROD) was represented as percentile values of control group (*n* = 5 per group). (**A**–**C**) Data are presented as mean value ± standard deviation and were analyzed using one-way ANOVA followed by Bonferroni’s post hoc test (^a^ *p* < 0.05, significantly different from the control group; ^b^ *p* < 0.05, significantly different from the vehicle group; ^c^ *p* < 0.05, significantly different from the CVE30 group).

Apoptosis and anti-apoptosis proteins such as Bax and Bcl-2 were measured by western blot analysis 3 days after ischemia. In the vehicle-treated ischemic group, Bax and Bcl-2 levels were significantly increased and decreased in the hippocampus to 402.6% and 36.0% compared to respective control group. In the CVE30-treated ischemic group, the proteins were similarly detected in the hippocampus compared to those in the vehicle-treated group. In the CVE300-treated ischemic group, Bax and Bcl-2 levels were significantly decreased to 65.6% and 131.5% of respective control group compared to those in vehicle- and CVE30-treated ischemic groups (Figure 4A).

MDA, IL-1β, IL-6, and TNF-α levels were assayed 6 h after ischemia to observe lipid peroxidation and inflammation in the hippocampus. In the vehicle-treated ischemic group, MDA, IL-1β, IL-6, and TNF-α levels were significantly increased to 210.0%, 545.7%, 327.0%, and 700.1%, respectively, in the control group. In the CVE30-treated ischemic group, MDA, IL-1β, IL-6, and TNF-α levels were similar to those in the vehicle-treated group. In the CVE300-treated ischemic group, IL-1β, IL-6, and TNF-α levels were significantly lower (139.5%, 186.3%, 193.0%, and 383.5% of the control group, respectively) compared to the levels in the vehicle-treated ischemic group (Figure 4B).

The morphology of microglia was visualized by immunohistochemical staining for ionized calcium-binding adapter molecule 1 (Iba-1) 4 d after ischemia. In the control group, Iba-1-immunoreactive microglia had a small cytoplasm with thin processes (resting microglia). In the vehicle- and CVE30-treated ischemic groups, Iba-1 immunoreactive microglia were abundantly observed in the stratum pyramidale of the CA1 region. They displayed round cytoplasm with no or less developed processes (phagocytic microglia). In these groups, Iba-1 immunoreactivity was significantly increased to 706.0% and 659.4% in the control group. In the CVE300-treated ischemic group, some Iba-1 immunoreactive microglia had hypertrophied cytoplasm with thickened processes (activated microglia). In this group, Iba-1 immunoreactivity was significantly decreased to 140.1% in the control group compared to the vehicle-treated ischemic group (Figure 4C).

**Figure 5:**
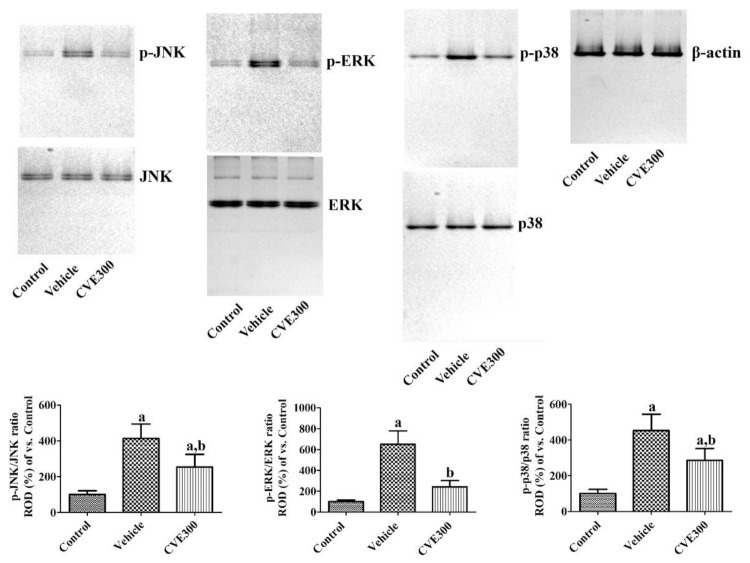
*Cissus verticillata* extract (CVE) ameliorates ischemia-induced phosphorylation of MAPKs. One day after ischemia, phosphorylation of MAPKs was assessed in the hippocampus of control, vehicle, and CVE300 groups by western blot for p-JNK, JNK, p-ERK, ERK, p-p38, and p38. Data are presented as mean value ± standard deviation and were analyzed using one-way ANOVA followed by Bonferroni’s post hoc test (^a^ *p* < 0.05, significantly different from the control group; ^b^ *p* < 0.05, significantly different from the vehicle group).

Mitogen-activated protein kinase (MAPK) levels were assessed in the hippocampus 1 d after ischemia. In the vehicle-treated ischemic group, the ratios of phosphorylated c-Jun N-terminal kinase (p-JNK)/JNK, phosphorylated extracellular signal-regulated kinase 1/2 (p-ERK)/ERK, and phosphorylated p38 (p-p38)/p38 were significantly increased to 413.9%, 650.7%, and 452.9%, respectively. In the CVE300-treated ischemic group, the ratios of p-JNK/JNK, p-ERK/ERK, and p-p38/p38 were lower (253.9%, 242.1%, and 285.9%, respectively) than in the vehicle-treated ischemic group (Figure 5).

## 3. Discussion

Lipid peroxidation and inflammation are major cell death mechanisms involved in oxidative and ischemic damage. *Cissus* spp. have antioxidant functions in various disease models [2,30,31,32,33,34,35]. In the present study, we validated the cellular toxicity of CVE in HT22 hippocampal cells based on the cell viability, ROS formation, and DNA fragmentation. We selected the optimal concentration of 50 μg/mL, which did not show significant changes in cell viability, ROS formation, and DNA fragmentation in HT22 cells. The neuroprotective effects of CVE against H_2_O_2_-induced oxidative damage were assessed in HT22 cells. Exposure to 1 mM H_2_O_2_ significantly increased ROS formation, DNA fragmentation, and cellular toxicity in HT22 cells. These results were consistent with previous studies showing that exposure to 1 mM H_2_O_2_ causes approximately 50–60% neuronal death in HT22 cells within 24 h after treatment [36] as well as increases ROS production [3,37] and Bax/Bcl-2 ratio [3,38]. In addition, we previously demonstrated ROS formation, DNA fragmentation, and cellular toxicity in HT22 cells after H_2_O_2_ treatment [31,32]. In the present study, treatment with 50 μg/mL CVE significantly ameliorated H_2_O_2_-induced cellular changes (ROS formation, DNA fragmentation, and cell death) and the Bax/Bcl-2 ratio in HT22 cells. To the best of our knowledge, this is the first study to show that CVE prevents neuronal damage induced by H_2_O_2_ in HT22 cells, although other *Cissus spp.* (*C. quadrangularis* extract) can lower H_2_O_2_-induced DNA damage and ROS formation in ECV304 human endothelial cells in a concentration-dependent manner [2]. However, *C. quadrangularis* extract facilitates apoptosis in A431 skin cancer cells by increasing the Bax/Bcl-2 ratio [39].

We also validated the in vivo neuroprotective potential of CVE in an ischemic model using gerbils. Transient forebrain ischemia in gerbils induces hyperlocomotor activity with a peak at 24 h after ischemia due to functional damage in the hippocampus [7,8]. We observed significant attenuation of ischemia-induced hyperlocomotion and neuronal damage in the hippocampal CA1 region at 300 mg/kg, but not 30 mg/kg, CVE treatment 1 and 4 d after ischemia. These results suggest that 300 mg/kg CVE has the potential to ameliorate neuronal damage induced by ischemia. A previous study showed anxiolytic and anticonvulsant effects of 300–1000 mg/kg CVE in mice [29]. In addition, CVE treatment significantly reduced the ischemia-induced apoptosis and increased anti-apoptotic protein, consistent with in vitro study. To elucidate the possible mechanisms against ischemic damage in the gerbil hippocampus, we focused on oxidative stress and inflammatory responses in the hippocampus after ischemia, because several studies have demonstrated that CVE has anti-inflammatory activity [28,40], and inflammation is one of the main pathways for neuronal death after ischemia in the gerbil hippocampus [41,42]. Overexpression of ROS induced by ischemia/reperfusion causes lipid peroxidation of polyunsaturated fatty acids in the neuronal membrane and disrupts the cell membrane [43].

In the present study, transient ischemia significantly increased lipid peroxidation and pro-inflammatory cytokines IL-1β, IL-6, and TNF-α in the hippocampus 6 h after ischemia. Treatment with 300 mg/kg CVE significantly decreased ischemia-induced lipid peroxidation and pro-inflammatory cytokine release in the hippocampus. In addition, microglial activation induced by ischemia was markedly decreased by treatment with 300 mg/kg, but not 30 mg/kg, CVE 4 d after ischemia. Several studies have demonstrated that *treatment with C. quadrangularis* mitigates lipid peroxidation in ovariectomized bone tissue [33], pilocarpine-induced epileptic hippocampus [34], and nicotinamide and streptozotocin-induced diabetic liver [32]. *C. quadrangularis* extract inhibits IL-1β induced inflammatory responses in chondrocytes [44]. However, few studies have reported the effects of CVE on lipid peroxidation and inflammation in the brain. CVE inhibits inflammation in animal models of edema in mice [28,40] and rats [40]. Transient forebrain ischemia increases the activity of the JNK, ERK, and p38 MAPKs by phosphorylation in the gerbil hippocampus [42,45,46]. In addition, MAPK inhibitors block inflammatory cytokine signaling [47,48] and treatment with the JNK inhibitor AS601245 protects neurons from ischemic cell death [49]. Consistent with previous studies [42,45,46], we observed significant increases in the ratios of p-JNK/JNK, p-ERK/ERK, and p-p38/p38 in the hippocampus 1 d after ischemia. Treatment with 300 mg/kg CVE significantly attenuated the increase in phosphorylation of JNK, ERK, and p38 in the hippocampus.

We demonstrated the neuroprotective effects and possible mechanisms (anti-inflammatory and MAPK inhibition) of CVE against oxidative damage in HT22 hippocampal cells and ischemic damage in the gerbil hippocampus. Several studies have revealed the ingredients of CVE, such as quercetin and kaempferol [28], *C. quadrangularis* extract, such as quercetin and resveratrol [2], and *C. repens* extracts, such as ursolic acid and lupeol [50]. In addition, a study demonstrates aerial part of *C. verticillata* has kaempferol, quercetin, 7,3′,4′-trihydroxyflavone, lanceolatin B, medicarpin, isoliquiritigenin, pongamol, and other chemical components [51]. Quercetin protects neurons from H_2_O_2_-induced cell damage in SH-SY5Y neuroblastoma cells [52] and protects against ischemic damage in gerbils [53,54]. Kaempferol reduces ischemic damage by inhibiting oxidative and inflammatory cascades [55]. Medicarpin has hydroxyl radical scavenging activity [56]. Isoliquiritigenin, ursolic acid, and lupeol alleviate the neuronal damage induced by ischemia [57,58,59]. Liquid chromatography–mass spectrometry studies need to be conducted to determine the functional components of CVE to show neuroprotective actions and mechanisms.

In conclusion, administration of CVE alleviates the neuronal damage induced by oxidative and ischemic stress in the hippocampus by decreasing the inflammatory responses and phosphorylation of JNK, ERK, and p38 in the hippocampus.

## 4. Materials and Methods

### 4.1. Preparation of CVE

The leaves of *C. verticillata* L. were taxonomically determined by Emeritus Prof. H.J. Chi, Seoul National University, South Korea. The fresh leaves were purchased from the local market in Ecuador collected in November 2016. The voucher specimen was deposited at the RIC herbarium at Hallym University (deposit number: HL201805). The leaves were dried at 40 °C for 48 h and dried leaves were homogenized for 2 min to the smallest possible particle size using a blender and extracted (500 g/5 L) by water extraction method (100 °C for 3 h) with a reflux condenser. The extract was freeze-dried at −50 °C temperature. The extract (75 g, 15% yield) was dissolved in ethanol (1 L) and centrifuged to the ethanol-soluble fraction, which (15 g, 20% yield from water extract) was evaporated to dryness by rotary evaporation as described previously [60].

### 4.2. In Vitro Toxicity of CVE to HT22 Cells

HT22 cells originating from the mouse hippocampus were purchased from ATCC (Manassas, VA, USA). They were cultured in Dulbecco’s modified Eagle’s medium as described previously [61]. CVE was dissolved in culture medium and HT22 cells were incubated with various concentrations (1–200 μg/mL) of CVE for 60 min. Thereafter, cells were harvested and assayed using the WST-1 assay kit (Sigma-Aldrich, St. Louis, MO, USA) to observe the conversion of tetrazolium salts into formazan by viable cells. Formazan fluorescence intensity was measured using a Fluoroskan ELISA plate reader (Labsystems Multiskan, Helsinki, Finland) as described previously [61]. Oxidative stress and DNA fragmentation induced by CVE was assessed 60 min after CVE treatment (50, 100, or 200 μg/mL). HT22 cells were incubated with 20 μM DCF diacetate (DCF-DA) to convert DCF-DA to DCF and cells were fixed for 3 h after 20 min of DCF-DA incubation. DNA fragmentation was visualized using a TUNEL staining kit (Sigma-Aldrich). DCF- and TUNEL-positive cells were observed by confocal fluorescence microscopy using an LSM 510 META NLO microscope (Carl Zeiss GmbH, Jena, Germany). DCF and TUNEL fluorescence intensities were measured using a Fluoroskan ELISA plate reader (Labsystems Multiskan).

### 4.3. Effects of CVE on H_2_O_2_-Induced Damage in HT22 Cells

Oxidative stress in HT22 cells was induced by exposure to 1 mM H_2_O_2_, and 50 μg/mL CVE was added to HT22 cells simultaneously with H_2_O_2_. Ten minutes after H_2_O_2_ and CVE treatment, HT22 cells were incubated with 20 μM DCF diacetate (DCF-DA) to convert DCF-DA to DCF. To measure ROS formation, cells were fixed for 3 h after H_2_O_2_ and CVE treatment. Cells were stained with a TUNEL staining kit (Sigma-Aldrich, St. Louis, MO, USA) to detect DNA fragmentation induced by H_2_O_2_. DCF- and TUNEL-positive cells were observed by confocal fluorescence microscopy using an LSM 510 META NLO microscope (Carl Zeiss GmbH, Jena, Germany). DCF and TUNEL fluorescence intensities were measured using a Fluoroskan ELISA plate reader (Labsystems Multiskan). Cell viability was measured using the WST-1 assay 5 h after H_2_O_2_ and CVE treatment, as described above. In addition, apoptosis and anti-apoptotic factors, including Bax and Bcl-2, were assayed by western blot. Briefly, cells were harvested 6 h after H_2_O_2_ treatment and lysed with ice-cold radioimmunoprecipitation assay buffer (Thermo Fisher Scientific, Waltham, MA, USA). Western blotting for Bax and Bcl-2 was performed as described previously [45]. Antibodies to Bax, Bcl-2, and β-actin were purchased from Abcam (Cambridge, UK) and were used at 1:2000 dilutions.

### 4.4. Experimental Animals

Male Mongolian gerbils were purchased from Japan SLC Inc. (Shizuoka, Japan). The animal experiments were conducted according to ethical guidelines for the use of animals in research. The protocols were approved by the Institutional Animal Care and Use Committee (IACUC) of Seoul National University (SNU-170630-8-2). Forebrain ischemia was induced by the occlusion of both common carotid arteries in the neck region as described previously [41,61]. CVE (30 or 300 mg/kg) or vehicle (distilled water) was administered to gerbils using a feeding needle.

### 4.5. Motor Activity

One day after ischemic surgery, gerbils were freed from the shuttle box. Their activity was recorded using a model 106200 digital camera system (Basler, Ahrensburg, Germany) for 60 min. Activities that included travel distance and time in immobile/mobile phases were analyzed using XT14 software (Ethovision, Wageningen, The Netherlands).

### 4.6. Immunohistochemical Staining

Mature neurons and microglia were visualized by immunohistochemical staining for NeuN and Iba-1 as described previously [61]. In brief, animals were re-anesthetized with isoflurane (Baxter, Deerfield, IL, USA) 4 d after ischemia, and transcardiac perfusion was performed with physiological saline and 4% paraformaldehyde. Thereafter, the brain was cryo-freezed and coronally sectioned (30 μm thick) based on gerbil atlas between 2.0 and 2.7 mm caudal to the bregma [62]. The antibodies used were mouse anti-NeuN antibody (1:1000; Merck Millipore, Temecula, CA, USA), rabbit anti-Iba-1 antibody (1:500; Fujifilm Wako Pure Chemical Corp., Osaka, Japan), goat anti-rabbit IgG (1:200, Vector, Burlingame, CA, USA), and goat anti-mouse IgG (1:200, Vector). Finally, immunoreactive signals were visualized by reaction with 3,3-diaminobenzidine tetrachloride (Sigma-Aldrich).

NeuN-immunoreactive nuclei were counted in the hippocampal CA1 region using OPTIMAS software (version 6.5; CyberMetrics^®^ Corporation, Phoenix, AZ, USA). Iba-1 immunoreactivity was assessed based on the intensity and pixel number of immunoreactive signals using ImageJ software (version 1.80; National Institutes of Health, Bethesda, MD, USA) as described previously [61].

### 4.7. ELISA

Pro-inflammatory cytokines IL-1β, IL-6, and TNF-α were measured in the hippocampus based on ELISA 6 h after ischemia, when the cytokines showed significantly higher levels compared to their respective sham group. Commercial IL-1β, IL-6, and TNF-α assay kits were purchased from R&D Systems (Minneapolis, MN, USA). The cytokine levels were calculated from standard curves from their standard solutions.

### 4.8. Western Blot

Apoptosis/anti-apoptosis were validated by western blot analysis for Bax and Bcl-2 in the hippocampus 3 days after ischemia, because the protein levels were significantly changed 3 days after ischemia [63]. MAPKs such as JNK, ERK, and p38, as well as their phosphorylated forms were assessed by western blot analysis in gerbil hippocampus 1 d after ischemia as previously described [45], because the phosphorylation of MAPKs is significantly increased in the gerbil hippocampus 1–3 d after ischemia [46]. All antibodies for MAPKs were purchased from Cell Signaling Technology (Danvers, MA, USA) and were used at a dilution of 1:1000.

### 4.9. Statistical Analyses

Acquired data are presented as mean and standard deviation. Differences in means were statistically analyzed using one-way ANOVA followed by Bonferroni’s post hoc test using GraphPad Prism 5.01 software (GraphPad Software, Inc., La Jolla, CA, USA).

## Data Availability

The datasets and supporting materials generated during and/or analyzed during the current study are available from the corresponding author on reasonable request.

## Supplementary Materials

The following are available online at https://www.mdpi.com/article/10.3390/plants10061217/s1, Figure S1: Hematoxylin and eosin staining in the hippocampal CA1 and CA2 region in the sham-operated (control), ischemia-induced vehicle-treated (vehicle), ischemia-induced 30 mg/kg CVE-treated (CVE30), and ischemia-induced 300 mg/kg CVE-treated (CVE300) groups (*n* = 5 per group). SO, stratum oriens; SP, stratum pyramidale; SR, stratum radiatum. Scale bar = 50 μm.
