# Supplementary material for: Cissus verticillata Extract Decreases Neuronal Damage Induced by Oxidative Stress in HT22 Cells and Ischemia in Gerbils by Reducing the Inflammation and Phosphorylation of MAPKs
Source: Plants (Basel). 2021 Jun 15;10(6):1217. doi: 10.3390/plants10061217 (PMC8232592; doi:10.3390/plants10061217)
Supplement: Supplementary file 1 [file plants-10-01217-s001.zip › plants-1212588-supplementary.pdf]

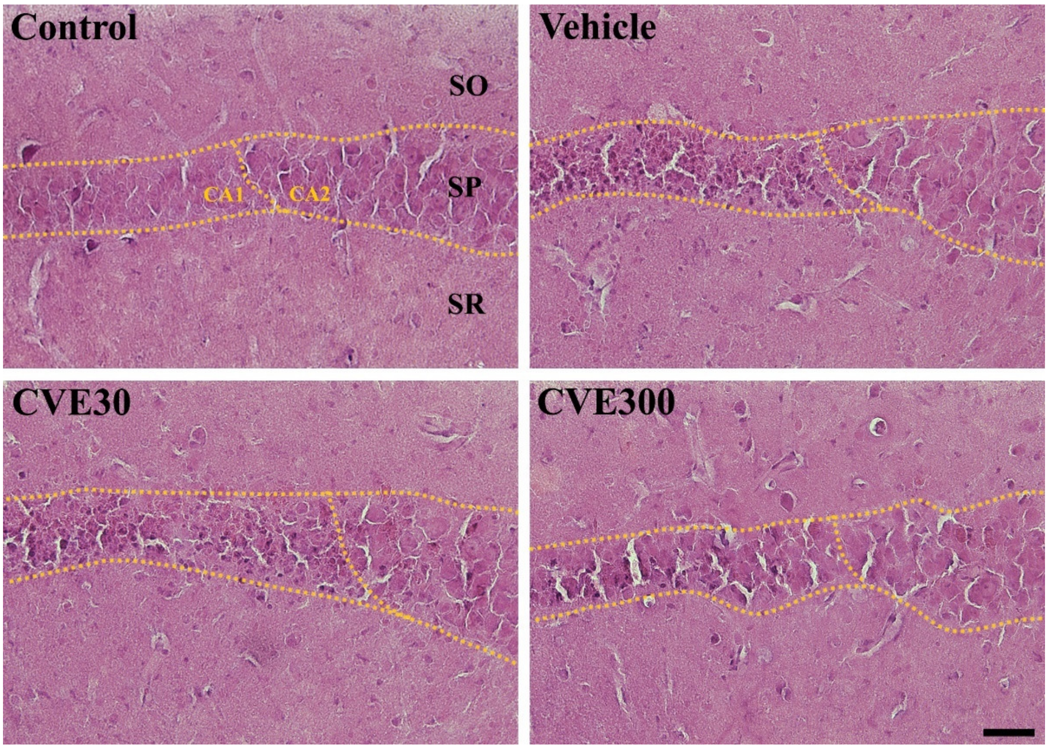

Supplementary Figure S1. Hematoxylin and eosin staining in the hippocampal CA1 and CA2 region in the sham-operated (control), ischemia-induced vehicle-treated (vehicle), ischemia-induced 30 mg/kg CVE-treated (CVE30), and ischemia-induced 300 mg/kg CVE-treated (CVE300) groups (*n* = 5 per group). SO, stratum oriens; SP, stratum pyramidale; SR, stratum radiatum. Scale bar = 50 μm.
